# 

**Published:** 1894-02

**Authors:** 


### CONTENTS.

ORIGINAL ARTICLES—

Cleft of the Hard and Soft Palates. By J. Ewing Mears, M. D., Philadelphia, Pa .... 59

Micro-Organisms and Cell Action in the Animal and Vegetable World—Pathologically considered. By A. Bethune Patterson, M. D., Atlanta, Ga .... 68

Acute Pneumonia in Childhood. By Thomas G. Southworth, M. D., New York .... 72

SOCIETY REPORTS—

Atlanta Society of Medicine .... 75

Clinical Society of Maryland .... 80

Academy of Medicine and Surgery, Richmond, Va .... 82

BOOK REVIEWS—

Duane's Students' Dictionary of Medicine. By Alexander Duane, M. D., New York .... 84

Minor Surgery and Bandaging. By Henry R. Wharton, M. D .... 84

SELECTIONS AND ABSTRACTS—

The Therapeutics of Glycozone, Composition and Characteristics. By Cyrus Edson, M. D., New York .... 85

A Plea for Aseptic Vaccine Virus and Aseptic Vaccination, with a Case in Point. By Rosa Engleman, A. B., M. D., Chicago .... 88

Permanganate of Potassium in the Treatment of Vulvo-Vaginitis in Little Girls, and Gonorrhœal Metro-Vaginitis in Women...... 90
The Radical Treatment of Stricture of the Rectum Viewed from the Standpoint of the Etiology. By John P. Bryson, M. D...... 91
The Relation between the Eye and the Female Genital Organs.... 94
Some Neuroses Associated with Menopause.......... 96
Anal Dilatation.......... 97
Hemorrhage from the Uterus.......... 98
The Relation of Syphilis to General Paresis.......... 98
In Gouty Rheumatism.......... 99
Golden Rules.......... 99

EDITORIAL—
Annual Meeting of the Medical Association of Georgia in Atlanta..100
The South Georgia Medical Association..........102
To Visit the South..........103
Editorial Notes..........105, 106

PRESCRIPTION DEPARTMENT..........108, 109
Burns—Neurasthenia—Tape Worm—For Fermentative Dyspepsia—Tonsillitis—Acute Tonsillitis—Obesity—Warts—Ovaritis—Whooping-Cough.

SPECIAL NOTES..........110–116

